# Do different incision techniques for implant surgery affect gingival papilla height around dental implants? A retrospective study of 115 cases

**DOI:** 10.1186/s12903-023-02828-z

**Published:** 2023-03-08

**Authors:** Chenchen Luo, Ming Chen

**Affiliations:** Beijing Stomatological Hospital, School of Stomatology, Capital Medical University, No.4, Tiantan Xili, Dongcheng District, Beijing, People’s Republic of China

**Keywords:** Dental implant, Dental papilla, Esthetics, Surgical incision

## Abstract

**Background:**

Surgical incision designs are considered closely correlated to gingival papilla of dental implants. This study aims to explore whether different incision techniques for implant placement and second stage surgery affect gingival papilla height.

**Methods:**

Cases using different incision techniques (intrasulcular incisions or papilla sparing incisions) between November 2017 and December 2020 were selected and analyzed. A digital camera was used to capture images of gingival papilla at different time points. Ratio of papilla height to crown length using different incision techniques were measured and statistically compared.

**Results:**

A total of 115 papillae (68 patients) were eligible according to the inclusion/exclusion criteria. The average age was 39.6 years. Decreased postoperative papilla height were observed after implant placement surgery in all groups without statistical difference. However, for second stage surgery, intrasulcular incisions lead to more atrophy in gingival papilla compared to papilla sparing incisions.

**Conclusions:**

Selection of incision techniques in implant placement surgery does not significantly affect papilla height. For second stage surgery, intrasulcular incisions significantly leads to more papilla atrophy compared with papilla sparing incisions.

*Trial registration* KQCL2017003.

## Background

Dental implants have been widely accepted as a major way to replace missing teeth and have been rapidly applied in partially dentate patients. For patients seeking implant therapy, the surgery is anticipated to be minimally invasive and less painful. Thus, methods for implant placement and second stage surgery should be carefully selected, aiming at reducing surgical complexity and patient visits. Also, the implant should be well-functioning, with satisfying esthetic effects and long-term periodontal stability.

Incision designs are considered closely correlated to papilla height, which have been investigated by several researchers in recent years. However, the results remain controversial. Some believe that the application of U-shape flap reduces the risk of gingival recession [1]. Animal study results [2] showed that sulcular incisions lead to more gingival recession compared with papilla sparing incisions, although recessions were observed in both groups. Anumala et al. [3] reported that incision type and flap design largely affect the soft tissue around dental implant, especially the preservation and reconstruction of gingival papilla. It is quite challenging to restore gingival papilla once they are destroyed in the implant placement surgery. However, some believe that the flap and incision designs do not significantly influence the gingival status. A randomized controlled trial by Girbés-Ballester et al. [4] reported that no significant difference in soft tissues were observed between *trapezoid* incisions (papilla sparing) and intrasulcular incisions. However, the sample size was limited. In short, the flap and incision design vary widely among present studies, with limited cases included. In this retrospective study, different incision designs are applied in the implant placement surgery and second-stage expose procedure. The corresponding papilla height at different time points are recorded and analyzed, aiming at optimizing incisions designs for implant surgery.

## Methods

A retrospective study was carried out to analyze the influence of different incision techniques on gingival papilla height. 68 patients (26 males and 42 females) aged 19–69 (average age 39.59 ± 12.89) who received single implant in the esthetic area at Beijing Stomatological Hospital between November 2017 and December 2020 were included in this study. A total of 115 papillae were eligible for the inclusion/exclusion criteria.

Inclusion criteria: 1. Dental implants functioning well for more than 6 months; 2. Single implant crown with natural adjacent teeth; 3. Thick gingival biotype with gingival thickness of > 1 mm; 4. The horizontal distance between implant and adjacent natural tooth is between 2.5 and 4 mm; 5. Normal occlusal relation; 6. Surgeries carried out by the same person; 7. Good compliance of patients who could undertake further consultation in due time.

Exclusion criteria: 1. Systemic diseases, such as hypertension, diabetes, heart disease, osteoporosis, hypo-immunity, etc.; 2. History of radiation therapy of head/neck; Acute inflammation in the implant area; 3. Poor implant orientation/location; 4. Uncontrolled periodontal disease. 5. Oral parafunctional habits, such as sleep bruxism and clenching; 6. Smoking (> 8 cigarettes per day).

According to Tettamanti et al*.* [5], two of the PES (pink esthetic score) indicis, mesial and distal height of the gingival papilla, were used as evaluation indicators. The ratios of papilla height using different surgical methods were recorded and analyzed. The papillae were divided into 3 groups (Table 1). For all cases adopting papilla sparing incisions in implant placement surgery, their papillae were also preserved in second stage surgery by papilla sparing techniques. The incision techniques were as follows: punch techniques [6], U-shaped technique [7], split finger technique [8], split pedicle roll envelope technique [9] (Figs. 8, 9, 10 and 11).

**Table 1:** Group classification and number of papillae included

	Group A	Group B	Group C
implant placement surgery	Intrasulcular incision	Papilla sparing techniques	Intrasulcular incision
Second Stage surgery	Intrasulcular incision	Papilla sparing techniques	Papilla sparing techniques
Number of papillae	34	18	63

**Fig. 1:**
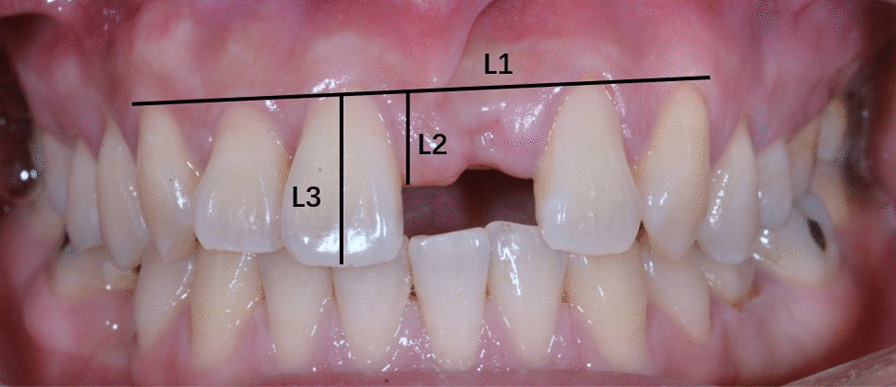
Measurement methods. A digital camera was used to take pictures perpendicular to the papilla and cervical 1/2 of the crown. Line 1: start from the most coronal point of marginal gingiva of uninjured adjacent teeth. Line 2: start from papilla, going in a direction perpendicular to line 1. Line 3: start from the most incisal point of the adjacent crown, in a direction perpendicular to line 1. Ratio of papilla height to crown length (V) = L2/L3

Measurement methods: A digital camera (Nikon D800, Nikon Corporation, Tokyo, Japan), was used to take pictures perpendicular to the papilla and cervical 1/2 of the crown. A line (Line 1) was made through the most coronal point of marginal gingiva of uninjured adjacent teeth (Fig. 1). Line 2 was made from papilla, going in a direction perpendicular to line 1. Line 3 was made from the most incisal point of the adjacent crown, in a direction perpendicular to line 1. The length of line 2 and line 3 were recorded as L2 and L3. Ratio of papilla height to crown length (V) = L2/L3. The values of V were calculated at different time points, including preoperatively (V1), 6 months after implant placement surgery (V2), 2 weeks after second stage surgery (V3), and immediately after crown placement (V4). When the papilla was located incisal to line 1, V was recorded as positive. As the papilla moved incisally, V became higher, presenting a more favorable esthetic outcome. When papilla moved coronally to line 1, V was recorded as negative, indicating severe papilla atrophy. All measurement analysis was completed by the same person.

ΔVα represented the changes in V before and after implant placement surgery (ΔVα = V1 − V2). ΔVβ represented the changes in V before and after second stage surgery (ΔVβ = V2 − V3); ΔVγ represented the change in V at crown placement compared with postoperative measurements (ΔVγ = V1 − V4). Differences were analyzed using ANOVA tests (Prism 9.0, GraphPad Software Inc., San Diego, CA) with 95% confidence interval. The methodology was reviewed by an independent statistician.

## Results

A total of 68 patients (115 papillae) were included in this study. All participants underwent second stage surgery. According to our results, the height of the gingival papilla around the implants decreased to varying degrees after implantation. The mean values of ∆Vγ (V1–V4) in all 3 groups were above zero, indicating extensive recession in gingival papilla after implantation (Fig. 4).

**Fig. 2:**
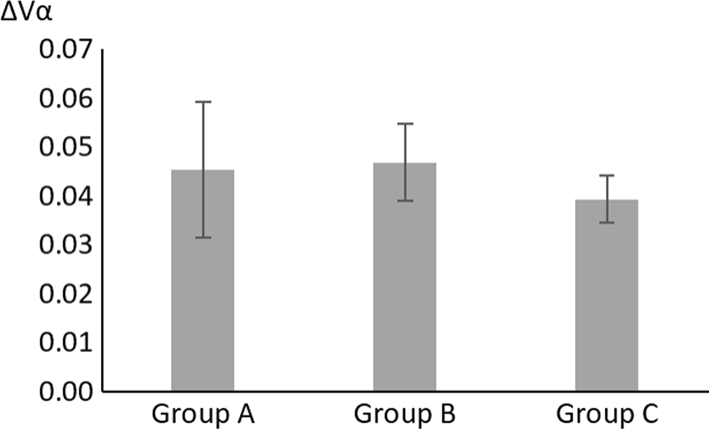
The changes in the value of V after implant placement surgery

No significant difference was found in papilla height after implant placement surgery among the 3 groups. Incisions in implant placement surgery results in decreased papilla height. The mean values of ∆Vα were 0.0453, 0.0469 and 0.0394 respectively for group A, B and C with no statistical difference (A vs. B, Kruskal–Wallis, Z = 0.3396, *P* > 0.05) (B vs. C, Kruskal–Wallis, Z = 0.2895, *P* > 0.05) (A vs. C, Kruskal–Wallis, Z = 0.1017, *P* > 0.05) (Fig. 2, Table 2).

**Table 2:** Analysis of results of different groups at different time points

∆V		Values	Statistical analysis	
V1–V2	A	0.0453 ± 0.0140	A versus. B: *P* > 0.9999 A versus C: *P* > 0.9999 B versus C: *P* > 0.9999	Kruskal–Wallis test
	B	0.0469 ± 0.0163		
	C	0.0394 ± 0.0093		
V2–V3	A	0.0827 ± 0.0138	A versus B: *P* < 0.0001 A versus C: *P* = 0.0002 B versus C: *P* = 0.2649	Kruskal–Wallis test
	B	− 0.0036 ± 0.0078		
	C	0.0143 ± 0.0049		
V1–V4	A	0.1616 ± 0.0200	A versus B: *P* = 0.0017 A versus C: *P* = 0.0015 B versus C: *P* = 0.7772	Brown-Forsythe and Welch ANOVA tests
	B	0.0634 ± 0.0168		
	C	0.0796 ± 0.0079		

Results are presented as the mean ± standard error

**Fig. 3:**
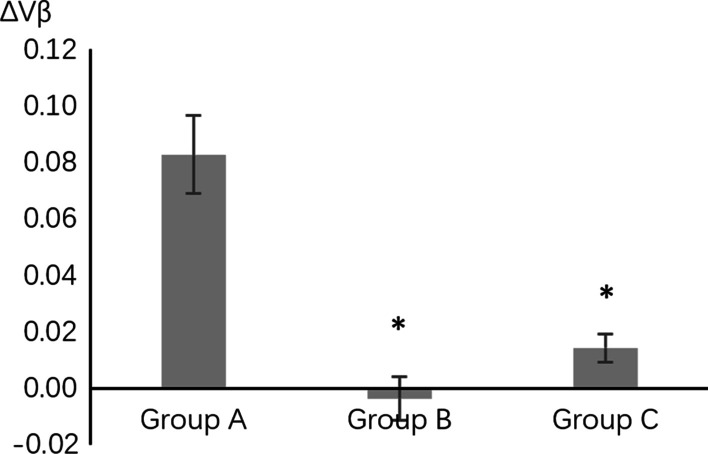
The changes in the value of V after second stage surgery. *significantly different compared with group A

Application of papilla sparing incision in second stage surgery significantly decreased postoperative papilla height. Results showed that the mean value of ∆Vβ (V2–V3) was 0.0827 for Group A using intrasulcular incisions in second stage surgery, while the value reduced significantly in the other 2 groups (A vs. B, Kruskal–Wallis, Z = 4.504, *P* < 0.05) (A vs. C, Kruskal–Wallis, Z = 4.029, *P* < 0.05), indicating that papilla sparing technique used in second stage procedure can avoid excessive gingival papilla absorption (Fig. 3).

**Fig. 4:**
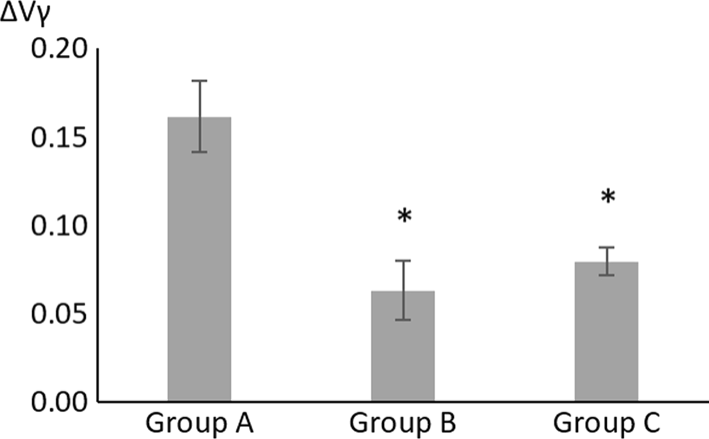
The changes in the value of V at crown placement compared with postoperative measurements. *significantly different compared with group A

**Fig. 5:**
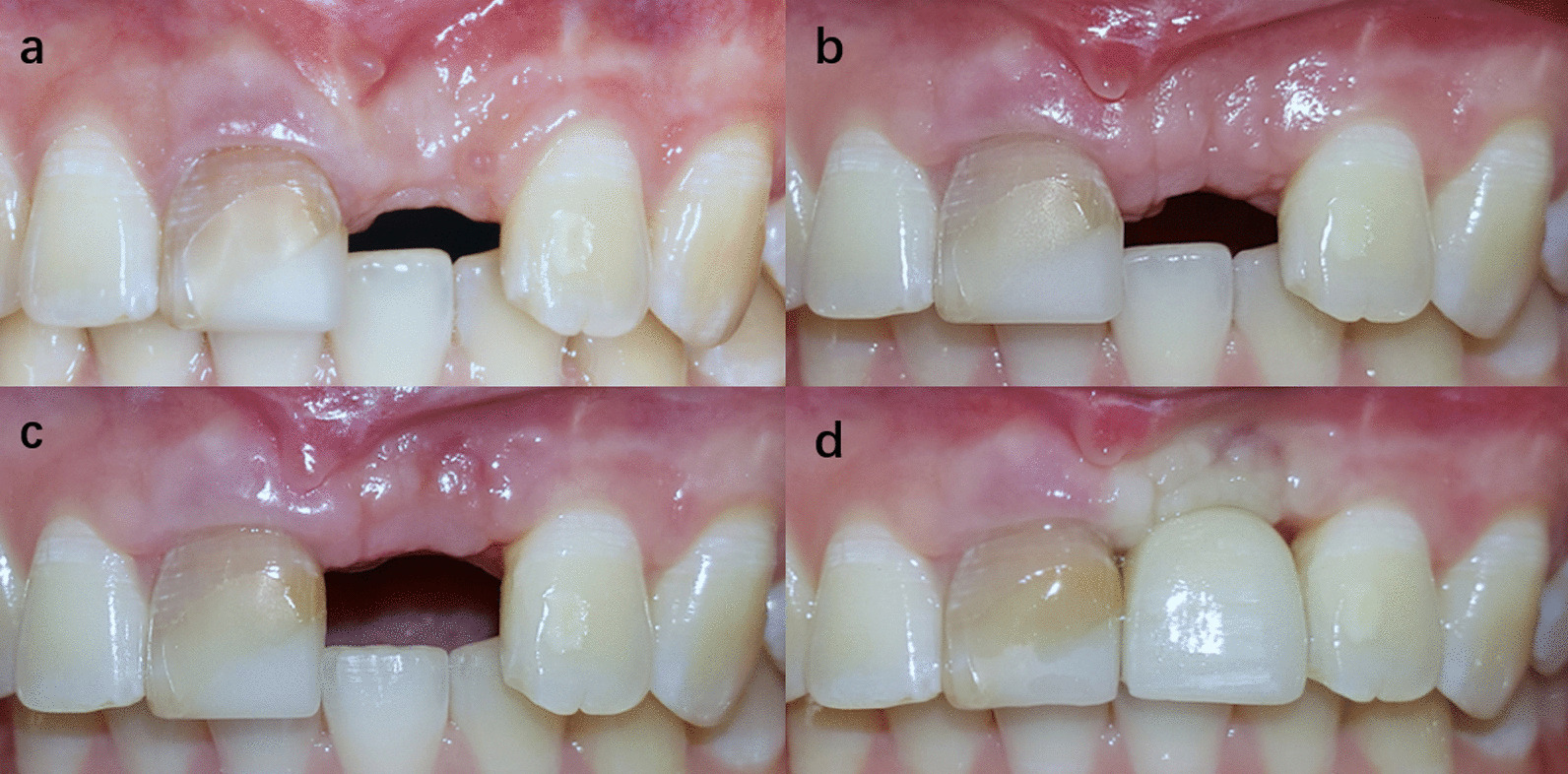
One of the cases of group A. Intrasulcular incisions were used in both implant placement surgery and second stage surgery. **a** Preoperative image. **b** 6 months after implant placement surgery. **c** 2 weeks after second stage surgery. **d** Immediately after crown placement

**Fig. 6:**
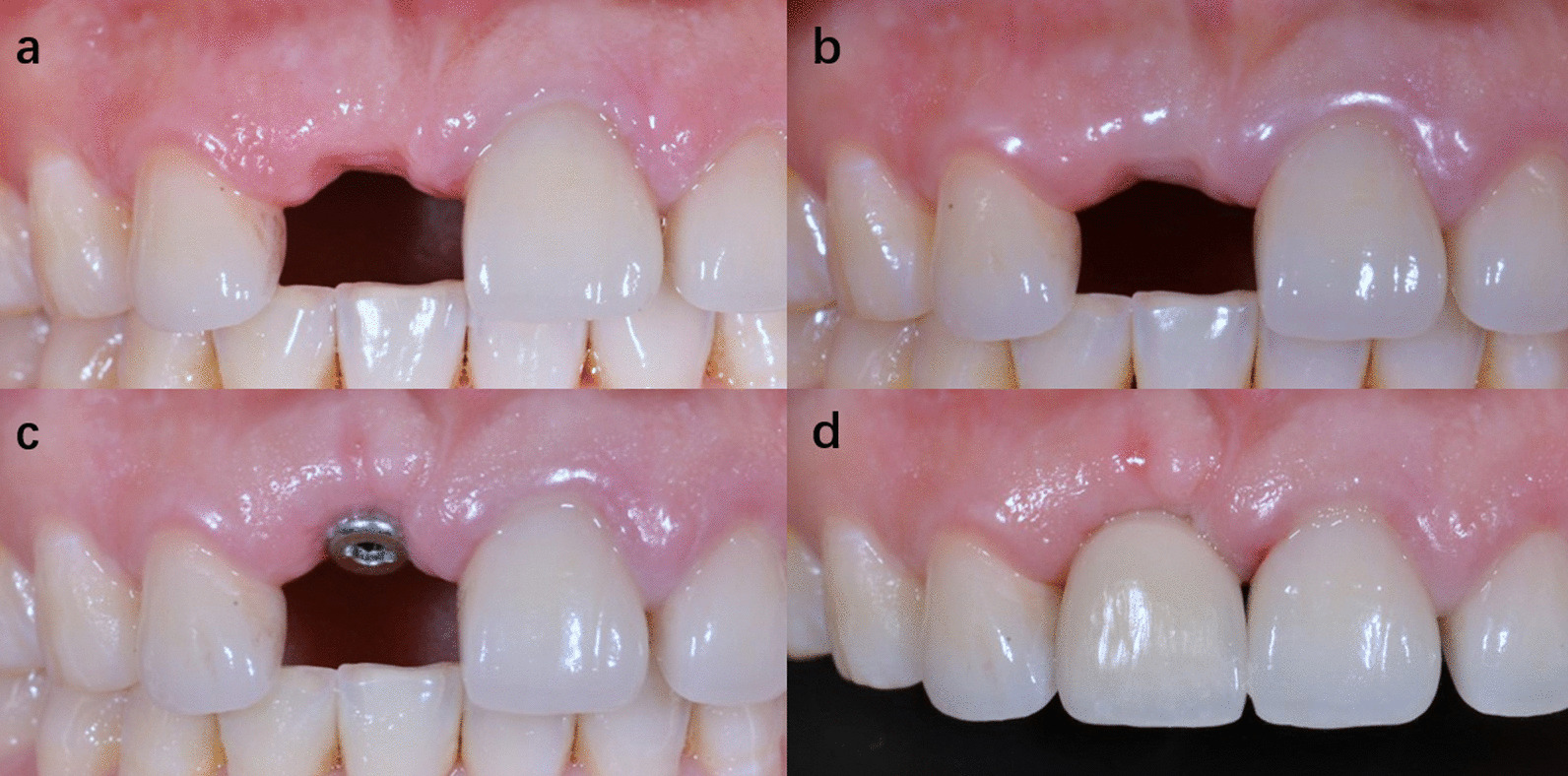
One of the cases of group B. Papilla sparing incisions were used in both implant placement surgery and second stage surgery. **a** Preoperative image. **b** 6 months after implant placement surgery. **c** 2 weeks after second stage surgery. **d** Immediately after crown placement

**Fig. 7:**
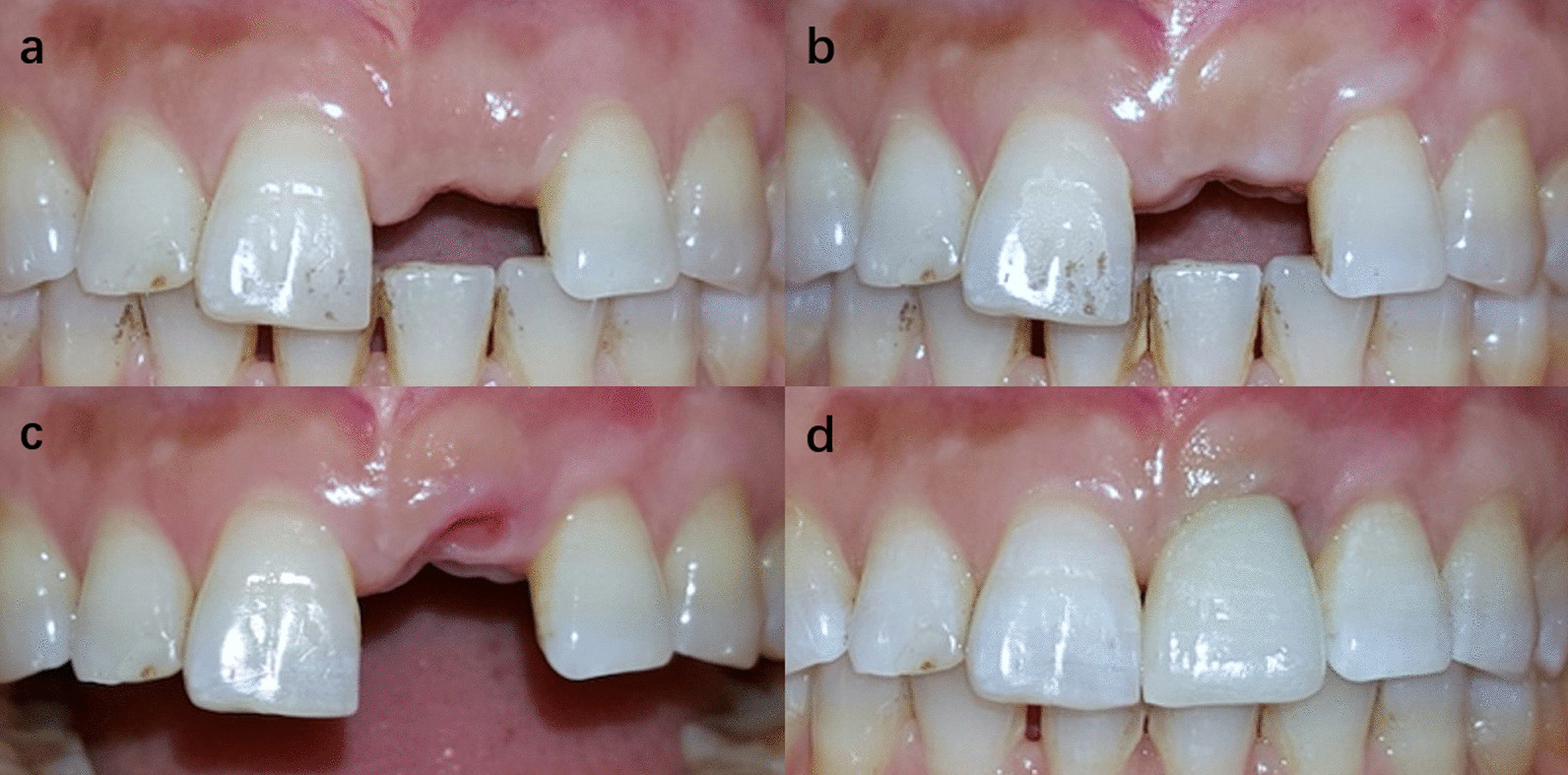
One of the cases of group C. Intrasulcular incisions were used in implant placement surgery and papilla sparing incisions were used in second stage surgery. **a** Preoperative image. **b** 6 months after implant placement surgery. **c** 2 weeks after second stage surgery. **d** Immediately after crown placement

Measurements were also made at implant placement (V4). Compared with V1 (preoperatively), the mean ∆Vγ (V1–V4) of group A was significantly higher than those of groups B and C (A vs. B, Brown-Forsythe and Welch ANOVA, *P* < 0.05) (A vs. C, Brown-Forsythe and Welch ANOVA, *P* < 0.05), indicating that intrasulcular incision in second stage surgery significantly leads to more papilla recession (Figs. 4, 5, 6 and 7). However, for implant placement surgery, no significant correlation was found between papilla height and selection of incision techniques (Fig. 2).

## Discussion

**Fig. 8:**
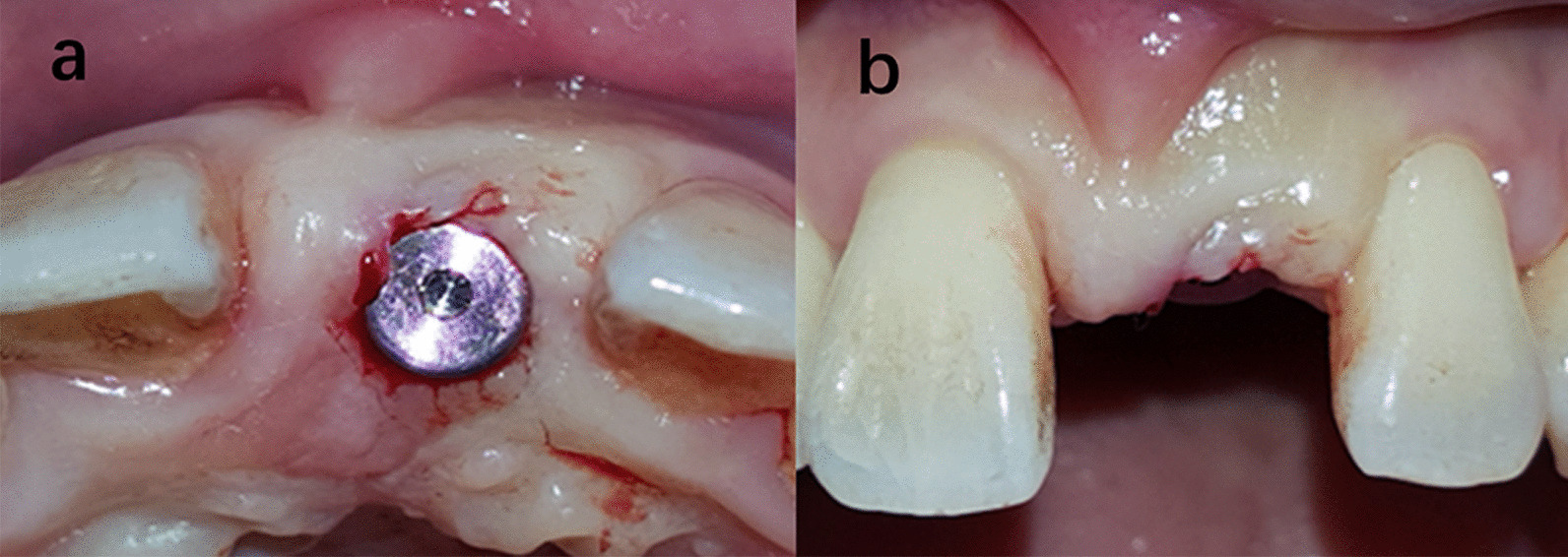
Punch techniques

**Fig. 9:**
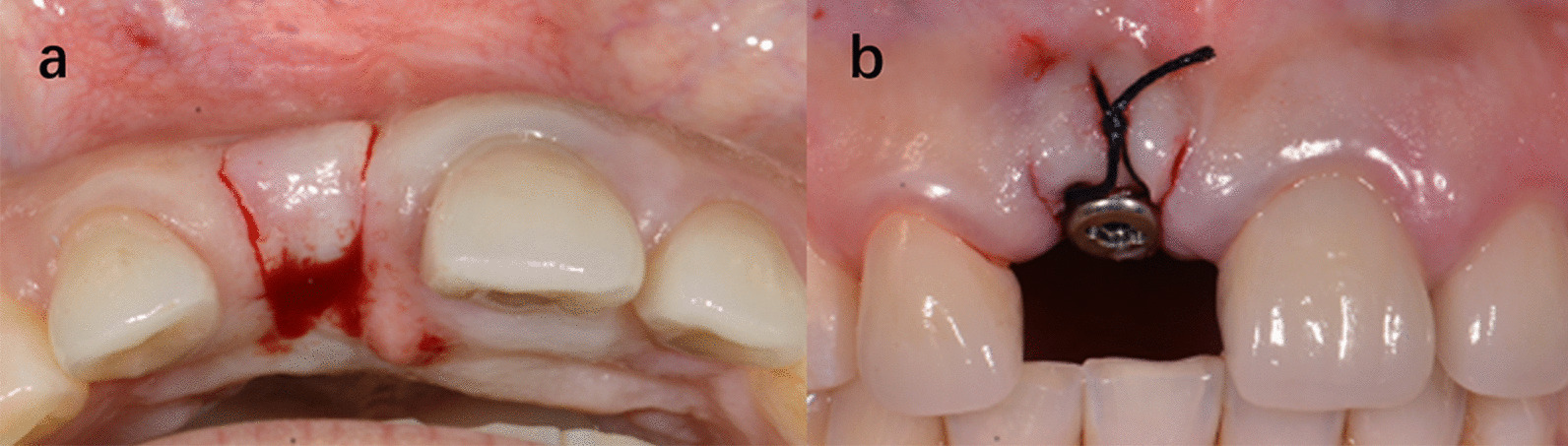
U-shaped technique

**Fig. 10:**
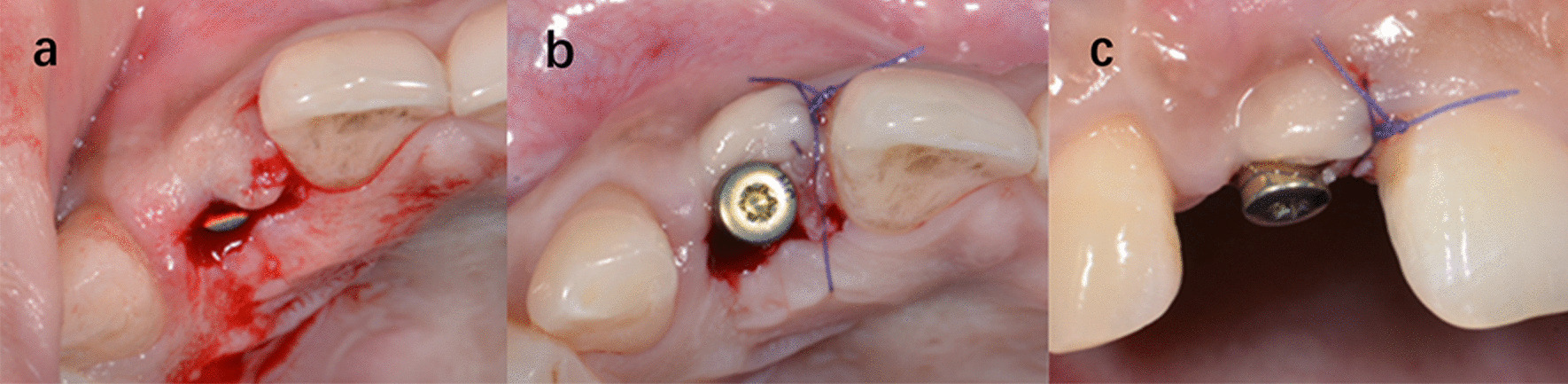
Split finger technique (mesial)

**Fig. 11:**
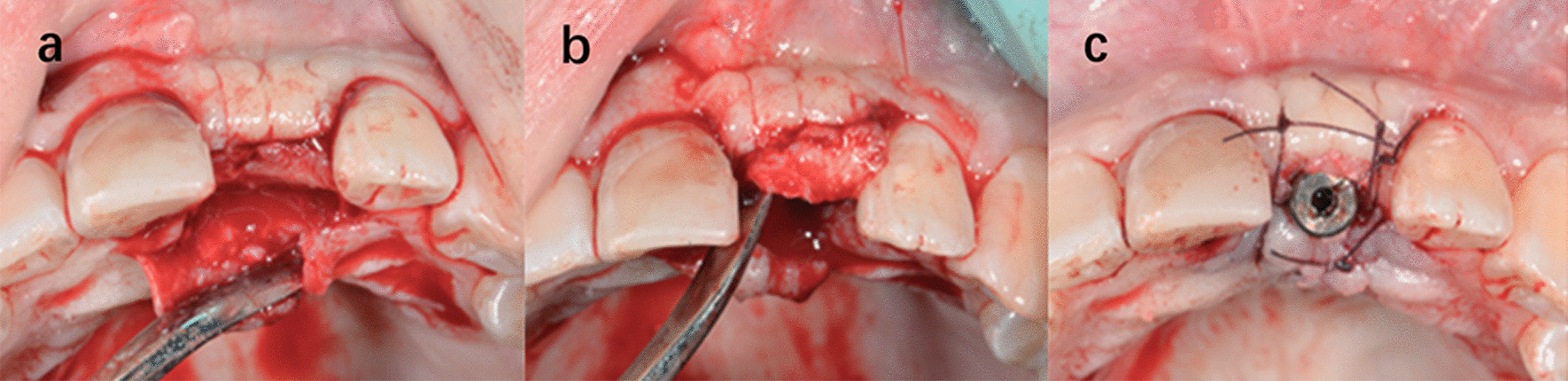
Split pedicle roll envelope technique

The objective of this retrospective study was to explore the influence of different incision designs on papilla height. Results showed that for second stage surgery, intrasulcular incisions result in enhanced papillary recession compared with papilla-sparing techniques. However, in implant placement surgery no statistical difference was observed (Figs. 8, 9, 10 and 11).

Achieving optimal gingival esthetics around dental implant is always considered challengeable. One of the factors that are heatedly discussed is incision designs in implant surgery. Castro-Calderón et al. [10] reveal that papilla base incision contributes to reducing buccal gingival recession. Jamal et al. [11] showed controversial results that the type of incisions does not influence papilla height or gingival levels. Thus, the impact of different incision techniques on papillary tissue is still worth investigating. Moreover, incision designs are closely related to surgical planning and outcomes. Improperly designed incisions lead to gingival scarring and incomplete wound closure, which may cause gingival esthetic problems. Thus, it is of great importance to explore the optimal incision techniques for dental implant treatment.

In this study, gingival recessions after crown restoration were observed in all groups compared with preoperative measurements, indicating that implant surgeries can cause damage to soft tissue and adversely affect gingival papilla height to various degrees. Implant surgery impair the stability of soft tissue, leading to lack of blood supply. Inadequate blood flow largely affects alveolar bone remodeling, and may further influence gingival contour. Therefore, to minimize surgical damage is of great importance in obtaining good esthetic outcomes.

Our results indicate that incision methods for implant placement surgery did not significantly affect papilla height. Results after implant placement surgery revealed that changes in gingival height among the 3 groups were not statistically different. Hutchens et al. [12] believed that the flapless approach in the maxillary anterior region reduce the surgical steps and maximize the esthetic effect of the anterior teeth during the implant placement surgery. The results in this study may be partially due to the tight sutures in implant placement surgery. Even conducted with guided bone regeneration (GBR), no postoperative wound dehiscence occurred after relaxation incisions. The closed gingival incision facilitates the reconstruction of blood vessels and ensures sufficient blood supply. Besides, 3 mm ± healing abutments were placed in most cases after implant placement surgery. The healing abutments support the gingiva and maintain the space for bone grafts [13]. In addition, some cases from the intrasulcular incision group used GBR, which may affect gingival height after implant placement surgery.

We also found that intrasulcular incisions in second stage surgery significantly decreased the gingival height. ΔVβ of group A was significantly higher than group B and C, indicating that minimizing surgical trauma in second stage surgery is of great importance in sustaining papilla height. Phadke et al. [14] believed that papilla sparing designs in second stage surgery may facilitate preserving the keratinized tissue around dental implant. For areas using intrasulcular incisions, placement of healing abutment may cause tensions on sutures, thus creating gaps between the incision ends of papillae. These gaps could only be closed by crawling replacement of soft tissue. Even when split-finger flap or split pedicle roll envelope technique were used to reconstruct gingival papilla, decreased papilla height was still observed. Besides, blood supply from periosteum, labial vessels and palatal vessels were damaged after intrasulcular incisions. Destruction of vessel networks lead to decreased nutrition, which results in alveolar bone resorption and papilla atrophy [15]. According to Zuhr et al. [16], papilla sparing incisions can ensure blood supply of gingival papilla, reduce invasion of periodontal microorganisms and minimize postoperative scar formation, which explains the results in this study.

Based on the results above, we recommend using papilla-sparing techniques in the second stage surgery. For implant placement surgery, papilla-sparing techniques are not necessary, especially when GBR is performed. Also, healing abutments of 3 mm height are recommended in the implant placement surgery, on the premise that the wound is completely closed.

There are several limitations in this study. The observation period in this study terminates at the time of crown placement. The papilla height of included cases may change with time, and we will continue to follow up. Also, this is a retrospectives study. Randomized controlled trials are needed to further investigate this issue.

### Considerations on experimental design

#### Gingival biotypes

According to Kan et al. [17], gingival thickness of > 1 mm are considered thick gingival biotypes and thickness ≤ 1 mm are considered thin gingival biotype. Studies have shown that different gingival biotypes respond differently to inflammation, surgery and injury, which have been considered an important factor in determining the esthetic outcomes of anterior teeth [18]. Thin gingival biotype is considered a high-risk factor for esthetic restoration. Mailoa et al. [19] believed that thin gingival biotypes easily lead to gingival recession. Also, *thin*- or *medium*-thickness are more prone to gingival recession than thick gingival biotypes [20]. Besides, the stable connections of desmosome and hemidesmosome beneath the keratinized mucosa can achieve excellent soft tissue sealing [21], block the accumulation of plaque, and buffer the stress caused by chewing or food impact. Thus, sufficient thickness of gingiva can reduce the occurrence of inflammation around the implant, prevent soft tissue shrinkage, and ensure the stability of dental implant [22].

In this study, thick gingival biotypes were selected to avoid the possible gingival recession caused by thin gingival biotypes. When the thickness of gingiva is maintained at a certain level, the gingival papilla become more stable, thus reducing its influence on experimental data.

#### Distance between implant and adjacent teeth

The distance between the implant and adjacent teeth is considered an important factor to influence papilla height. Romeo et al. [23] found that when the horizontal distance between the implant and the adjacent teeth is between 2.5 and 4.0 mm, gingival papilla was significantly present. Smaller interdental space often results in unsatisfactory gingival outcomes. Esposito et al. [24] conducted a 3-year follow-up study of 58 patients with implant restorations and found that small distance between the implant and adjacent teeth leads to decreased papilla height and alveolar bone level. Also, a full interproximal papilla is difficult to achieve when the distance is too large. Based on the evidence above, cases with interdental distance of 2.5–4 mm were included in this study.

#### Measurement methods

In this study, a digital camera was used capture images of gingival papilla of each patient. The pictures were taken at different time points during treatment. Camera should be parallel to the occlusal plane, and be pointed in a perpendicular angle to gingival papilla and cervical 1/2 of the buccal surface of teeth. Every picture should follow this protocol. All pictures were imported in a computer for detailed measurement, which is more recognizable and convenient, and can be measured repeatedly. It is believed that the error caused by photo shooting can be massively reduced by strictly following the protocol. Priest et al. [25] compared the PES values of two pictures taken on the same patient, and results showed excellent consistency. On the contrary, intraoral measurement via periodontal probes often leads to compromised accuracy by using millimeter as a measurement unit. Therefore, intraoral imaging is considered an accurate and objective way to evaluate soft tissue around single-tooth implant, which is also suitable for PES assessment.

#### Evaluation methods

There are several indices available to evaluate gingival esthetics. For example, in 2005, Fürhauser et al. [26] suggest using Pink Esthetic Score (PES) to evaluate soft tissue, which contains 7 observation indexes, including mesial papilla, distal papilla, soft-tissue level, soft-tissue contour, alveolar process deficiency, soft-tissue color and texture. In 2005, Meijer et al. [27] proposed Implant Crown Aesthetic Index (ICAI), including 9 parameters to evaluate both implant-supported single crowns and adjacent soft tissues. In 2009 Belser et al. [28] proposed the red and white aesthetic index PES/WES. A study conducted by Hof et al. [29] evaluated 8 indices including Papilla Index [PI], Pink Esthetic Score [PES], Implant Crown Aesthetic Index [ICAI], Pink and White Esthetic Score [PES/WES], Complex Esthetic Index [CEI], Implant Aesthetic Score [IAS], Subjective Esthetic Score [SES], and Rompen Index. Among the parameters above, pink esthetic scores (PES) showed significantly higher inter-rater reproducibility and intra-rater reproducibility (inter r = 0.66, intra r = 0.88), which is also proved by other researchers [30]. Evaluation indices such as color and alveolar process morphology presented lower scores. This may be because that these variables are more susceptible to subjective factors and therefore showed higher variability. In this study, gingival papilla was used as an indicator, which is intuitive and quantifiable. Instead of using the scoring system, we did accurate measurement and use ratios to represent the papilla height. This approach precisely revealed the changes in papilla height using different incision methods, which is more objective and accurate.

Jemt [31]. proposed papilla index score of 0, 1, 2, 3, 4 to describe papilla height after implant restoration. However, the papilla status before restoration cannot be evaluated, and the hierarchical value used in this system may lead to compromised accuracy. Gressberg [32] introduced a fixed and reproducible reference line, which goes through the highest points of gingival margin of uninjured adjacent teeth. The distance from the fixed line to the most coronal point of gingival papilla were measured in photographs. However, the magnification needs to be corrected each time. Besides, the specific figures in the intraoral measurement can only accurate to 0.1 mm, with relatively larger errors and lower repeatability. In this study, measurements on computers are more precise with perfect repeatability. Also, with the introduction of ratios, magnifications no longer need to be corrected.

## Conclusion

The incision designs for implant surgery have a certain impact on the esthetics of the gingiva. This study reveals that selection of incision techniques in implant placement surgery does not significantly affect papilla height. However, for second stage surgery, intrasulcular incisions significantly leads to more papilla atrophy compared with papilla sparing incisions.

## Data Availability

All data generated or analyzed during this study are included in this published article.

## Abbreviations

GBR: Guided bone regeneration
PES: Pink esthetic score
PES/WES: Red and white aesthetic index
PI: Papilla index
ICAI: Implant crown aesthetic index
CEI: Complex esthetic index
IAS: Implant aesthetic score
SES: Subjective esthetic score
